# Frosty Weather

**Published:** 1867-12

**Authors:** 


FROSTY WEATHER.—Few have failed to observe what a vigor and elasticity are imparted to both mind and body by a frosty atmosphere, and what a loss of all these there is in a hot summer day; this is probably owing to the fact that at noon of any clear frosty day in winter, there is ten times as much elasticity in the air as there is at any noon of summer; hence to all invalids, the days most valuable for exercice are those of frosty weather, and those least beneficial are where it is warm or thundery; hence every hour of daylight spent in the open air in frosty weather in some kind of out-door activities is that much gain to the vitality of the system, imparting vigor to the mind, elasticity to the body, and elevation to the moral feelings and power of the man.

